# Doege-Potter syndrome: When seizures and hypoglycemia collide

**Published:** 2018-04-04

**Authors:** Marianna Riolo, Valentina Arnao, Fabio Giacalone, Roberto Citarrella, Daniela Cabibbi, Massimo Cajozzo, Paolo Aridon

**Affiliations:** 1 Department of Experimental Biomedicine, Neuroscience Clinic, University of Palermo, Palermo, Italy; 2 Biomedical Department of Internal and Specialized Medicine (DIBIMIS), University of Palermo, Palermo, Italy; 3 Department of Science for the Promotion of Mother and Child Health, University of Palermo, Palermo, Italy; 4 Department of Oncological and Stomatological Surgical Disciplines, University of Palermo, Palermo, Italy

**Keywords:** Seizures, Hypoglycemia, Solitary Fibrous Tumor of the Pleura, Reactive Hypoglycemia, Insulin-Like Growth Factors, Electroencephalography

An estimated 150000 adults present annually with an unprovoked first seizure in the United States, with 25% of new onset seizures occurring in individuals over the age of 65 years.^1^ In this group, the correct diagnosis cannot be so straightforward, due to the broad spectrum of conditions causing and mimicking it. Between these, hypoglycemia acts an important role with its chameleonic clinical presentation; for this reason, an accurate diagnostic work-up is needed to correctly categorize and detect causes that can provoke it. One of these is non-islet cell tumor-induced hypoglycemia (NICTH), also known as Doege-Potter syndrome (DPS), a rare paraneoplastic syndrome seen in association with solitary fibrous tumors of the pleura (SFTP).^2^ Here, we chronicle a case of this rare condition. 

A 76-years-old Caucasian man presented to the Emergency Department of our hospital complaining of repeated episodes of loss of consciousness with mild traumas and incontinence. Vital signs evaluation and laboratory tests were normal, except for blood glucose level of 39 mg/dl. His medical history revealed a mild hypertension, under medical treatment, without a history of diabetes. A neurologic evaluation was performed and it showed a general slowdown, above all in reaction and language, a disinhibited personality, fine tremor of upper limbs, and clumsiness on bilateral manual dexterity.

The patient was admitted to neurological ward, and during hospitalization, he had some episodes of asymptomatic hypoglycemia, as well as phases of agitation associated with clonic movements in limbs, in conjunction with hypoglycemia (the lowest glucose level recorded was 31 mg/dl). For this reason, levetiracetam, carbamazepine, and a continuous infusion of 10% dextrose were administered, with clinical improvement. Brain imaging [computed tomography (CT) scan and magnetic resonance imaging (MRI)] and cardiological evaluation were normal; an interictal electroencephalography (EEG) recorded was globally slowed without the presence of epileptiform discharges. In addition, the combined dosage of extemporaneous glucose, insulin, C-peptide, and growth hormone (GH) levels were evaluated during a hypoglycemic crisis, revealing low levels of insulin and C-peptide, with GH in ranges. Oral glucose tolerance test was also performed, with a blood glucose level of 210 mg/dl at time 120 minutes, consistent with diabetes mellitus type II. During this test, insulin and C-peptide remained suppressed too.

In the suspect of a paraneoplastic origin of symptoms, a thorax and abdomen CT scan was performed showing the presence, on the right part of the thorax, of a voluminous, heterogeneous mass of diaphragmatic origin, compressing the adjacent lung, associated with homolateral pleural effusion. For this reason, a percutaneous needle CT-guided biopsy was performed, showing morphological cytoarchitecture and immunohistochemical features (positivity for CD34 and vimentin) typical of a solitary fibrous tumor. Consequently, the patient underwent to thoracotomy with mass excision (Figure 1), which confirmed the diagnosis. 

**Figure 1:**
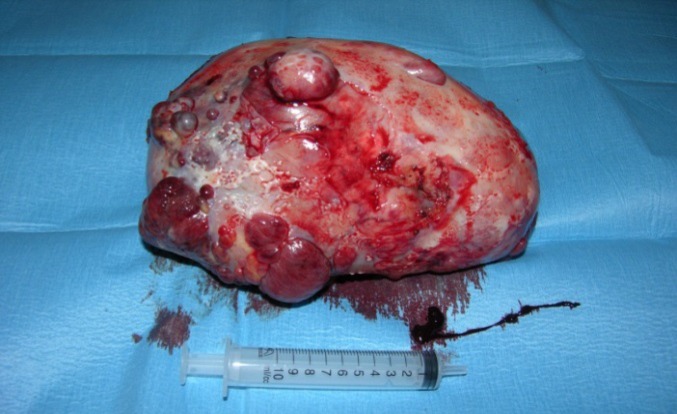
Voluminous and dishomogeneous mass after surgical removing

After the surgery, the patient did not experienced any other episode. 

SFTPs are rare spindle cell neoplasm arising from submesothelial mesenchymal layer;^2^ generally, 80% of SFTPs are benign. When they manifest with refractory hypoglycemia, DPS occurs. This paraneoplastic syndrome is an uncommon complication, occurring in less than 5% of SFTP cases;^2^ significant hypoglycemia is related to SFTP in approximately 4% of cases. It is the consequence of tumor production of a prohormone form of insulin-like growth factor II (IGF-II), which is responsible of the malignant and refractory hypoglycemia. Multiple mechanisms are involved, as increased glucose uptake by insulin-sensitive tissues and by the tumor itself, decreased hepatic glucose production, and suppressed insulin and C-peptide levels.^3^

Our case highlights the importance of considering uncommon causes in a healthy-appearing patient presenting with seizure-like episodes and hypoglycemia, which can have a broad differential diagnosis. Moreover, the elderly have unique diagnostic challenges because of peculiarity in the clinical presentation, etiology, coexistence of co-morbidities, and cognitive difficulties.^4^ In our case, a normal finding in brain imaging excluded a structural lesion causing clinical events; moreover, personal history and EEG registration ruled out seizures. Otherwise, the coexistence of low glucose levels during clinical events, and resolution of symptoms after glucose administration (Whipple's triad) suggested hypoglycemia as the likely cause. In fact, at rest, the brain accounts for 60% of the total basal glucose consumption, with its limited glycogen reserves, so it is not surprising that some of the primary symptoms of hypoglycemia are neurologic.^5^ In our case, endocrinology tests were performed, revealing the low levels of serum insulin and C-peptide which did not suggested an exogenous or endogenous hypoglycemia, but an IGF-mediated one. 

In conclusion, our case describes the occurrence of a rare pathological entity presenting with a rarer clinical syndrome. Nowadays, physicians have to face with complicated scenarios, and an open-minded thinking is mandatory in the differential diagnosis. This is the reason why if an elderly person presents with frequent falls, “strange movements”, or blackouts, it is necessary to obtain detailed history, to perform a complete neurological and general examination, to consider neurological, cardiologic, and internist causes, and to hold a holistic approach.

## Notes:

**How to cite this article:** Riolo M, Arnao V, Giacalone F, Citarrella R, Cabibbi D, Cajozzo M, et al. Doege-Potter syndrome: When seizures and hypoglycemia collide. Iran J Neurol 2018; 17(2): 86-8.
